# Perceived Loudness of Self-Generated Sounds Is Differentially Modified by Expected Sound Intensity

**DOI:** 10.1371/journal.pone.0127651

**Published:** 2015-05-18

**Authors:** Daniel Reznik, Yael Henkin, Osnat Levy, Roy Mukamel

**Affiliations:** 1 School of Psychological Sciences, Tel Aviv University, Tel Aviv, Israel; 2 Sagol School of Neuroscience, Tel Aviv University, Tel Aviv, Israel; 3 Department of Communication Disorders, Sackler Faculty of Medicine, Tel Aviv University, Tel Aviv, Israel; 4 Hearing, Speech, and Language Center, Sheba Medical Center, Tel Hashomer, Ramat Gan, Israel; UNLV, UNITED STATES

## Abstract

Performing actions with sensory consequences modifies physiological and behavioral responses relative to otherwise identical sensory input perceived in a passive manner. It is assumed that such modifications occur through an efference copy sent from motor cortex to sensory regions during performance of voluntary actions. In the auditory domain most behavioral studies report attenuated perceived loudness of self-generated auditory action-consequences. However, several recent behavioral and physiological studies report enhanced responses to such consequences. Here we manipulated the intensity of self-generated and externally-generated sounds and examined the type of perceptual modification (enhancement vs. attenuation) reported by healthy human subjects. We found that when the intensity of self-generated sounds was low, perceived loudness is enhanced. Conversely, when the intensity of self-generated sounds was high, perceived loudness is attenuated. These results might reconcile some of the apparent discrepancies in the reported literature and suggest that efference copies can adapt perception according to the differential sensory context of voluntary actions.

## Introduction

Research over the last decades has demonstrated that physiological and behavioral responses to self-generated action consequences are modified relative to otherwise identical sensory input generated by an external source [1,2]. Thus, for example, physically identical sounds evoke different neural responses in auditory cortex and are perceived differently, depending on the sound-producing source (such as self or other) [3,4]. It is believed that the source of such modifications is an efference copy sent from the motor to sensory cortices in parallel to the motor commands relayed to the relevant effectors [5,6]. Such signals have been shown to facilitate, inhibit, or otherwise modify neural responses to sensory consequences of self-generated actions [7].

In humans, some studies report attenuated [8–11] and others report enhanced [12–15] neural and behavioral responses to self-generated stimuli. Studies reporting attenuated responses are compatible with a model in which the functional role of the efference copy is to dampen/cancel sensory feedback of self-generated action consequences [7,16]. This might have ecological importance in order to avoid desensitization of the sensory apparatus during action performance while maintaining sensitivity to external stimuli [17]. Another suggested functional role is correct attribution of the source of the sensory stimulus (self/other) [18]. Indeed dysfunction of such a dampening mechanism in the auditory domain, for example, was found in patients diagnosed with schizophrenia and has been suggested to drive auditory hallucinations and misattribution of sound source [19,20]. However studies reporting enhanced responses to self-generated sounds suggest that this canceling model cannot be effectively generalized.

In the auditory domain, studies reporting attenuation of perceived loudness for self-generated sounds typically used comparison tasks between self-generated and externally-generated sounds [4,21]. Conversely, using an auditory detection task, it has been reported that detection thresholds for self-generated sounds are actually lower (i.e. better detection) [14]. Since detection tasks present stimuli at near-threshold intensities and the studies using comparison tasks presented sounds well above hearing level (typically around 70 dBSPL), we hypothesize that the intensity of self-generated auditory consequences might be an important parameter governing the type of behavioral shift in perception (enhancement/attenuation relative to passive hearing). Specifically, we hypothesize that when the intensity of self-generated auditory consequences is low, their perceived loudness is enhanced and when the intensity of self-generated auditory consequences is high, their perceived loudness is attenuated.

## Material and Methods

### Participants

Thirty healthy, normal hearing subjects were recruited to the study. Data from one subject was excluded due to technical problems and data from an additional subject was excluded due to task performance which was 2.8 standard deviations above the group average, leaving data from 28 subjects (three left-handed; 10 males; mean age, 25.1, range 22 to 30 years). Subjects were compensated for their time and provided written informed consent to participate in the study. The study was approved by the ethical committee of Tel-Aviv University.

### Procedure, apparatus and stimuli

Subjects were seated in a soundproof chamber and auditory stimuli were presented to both ears via TDH-39 headphones by means of a GSI-61 audiometer. Visual instructions were provided by a computer screen located in front of the chamber's window. During each experimental trial, two consecutive 1kHz pure tones (duration 300 ms including linear rise/decay time of 25 ms) were presented, and subjects’ task was to report whether the first or second tone was louder. Unbeknownst to the subjects, in all experimental trials the intensity of the two tones was identical.

**Fig 1:**
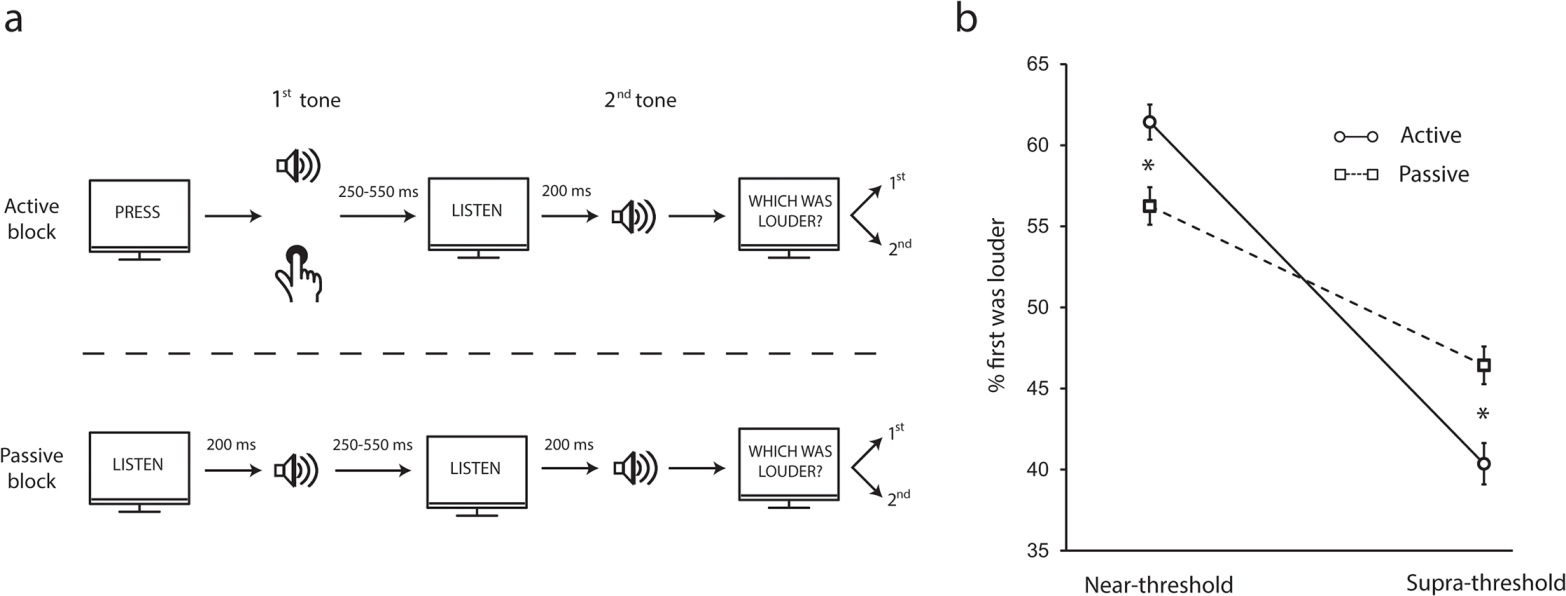
Experiment design and Results. **(a)** Subjects were presented with two tones and had to decide which tone was louder. In the active condition subjects triggered the first tone by a button press whereas the second tone was generated by a computer (top); in the passive condition, both tones were generated by a computer (bottom). **(b)** Mean % trials in which 1^st^ tone was reported being louder (error bars denote s.e.m. across 28 subjects). Perceived loudness of self-generated sounds is differently modified by the sound intensity (repeated measured ANOVA, p<0.05). The asterisks denote significant differences between corresponding active and passive conditions within each sound intensity (p<0.05 one-tailed paired t-test).

In the *active* condition, the word “PRESS” appearing on the computer screen cued the subjects to press a button with their right index finger which triggered the first tone. Following a variable interval between 250 and 550 ms (sampled from a uniform distribution for each trial) the word "LISTEN" appeared on the screen for 400 ms and served as a cue for the presentation of an identical second tone generated by the computer 200 ms after the visual cue onset. In the *passive* condition, both first and second tones were generated by a computer 200 ms following a visual cue ("LISTEN"), thus the appearance of all computer generated tones was always fully predictable by the visual cue. In both conditions, after presentation of the second tone, subjects reported which tone was louder by pressing one of two corresponding buttons with their left hand (Fig 1A). The percent of trials reported as "1^st^ tone louder^"^ was taken as the dependent measure in each subject.

Active/passive conditions were presented in separate blocks consisting of 50 experimental trials. To engage subjects' attention and to prevent response bias, each separate block of active and passive conditions contained 10 additional randomly interspersed oddball trials in which the intensity of the second tone was either 2dB higher (5 trials) or 2dB lower (5 trials) than the first tone.

Each pair of active/passive blocks was repeated twice, once using tones presented at supra-threshold intensity (75dBSPL) and once using tones presented at near-threshold intensity (5dB above individual subjects' threshold). Binaural auditory thresholds were measured for each subject using the "*1step up*, *2 steps down*" method [22] (starting at 5 dBHL in 5dB steps). Importantly, in post-experiment debriefing, subjects reported that in the near-threshold intensity both tones were distinguishably heard. The order of intensities (supra/near) and conditions within each intensity (active/passive) was randomized across subjects.

### Statistical analyses

In the active conditions, the first 30 experimental trials served as an acquisition phase during which the subjects learned to associate the button press with its auditory consequences [4,21]; the last 20 experimental trials were used for examining perceptual differences between the active and corresponding last 20 trials in the passive condition.

Effects of interest were tested using one-tailed paired t-tests and repeated measures analysis of variance (ANOVA) with two within subjects variables—condition (active\passive) and intensity (supra-\near-threshold). All tests were conducted using a significance level of α = 0.05.

## Results

The median binaural hearing threshold across subjects was -2.5dBHL; range -7.5 to 12.5dBHL. Subjects performed the comparison task adequately with 85.1±1.2% correct responses in oddball trials (mean ± standard error of the mean (s.e.m.) across subjects).

Repeated measures ANOVA revealed a significant interaction between condition (active/passive) and intensity (supra/near-threshold; F_(1,27)_ = 7.26, p<0.05, *η* ^2^_*p*_ = 0.21; Fig 1B). Follow-up examinations revealed that at near-threshold intensity, subjects perceived more often the first (self-generated) tone as being louder in the active compared with passive condition (mean percentage of trials ± s.e.m. across subjects, active condition: 61.4±2.1%, passive condition: 56.2±2.1%, *one-tailed paired t-test*, n = 28; t_(27)_ = 1.85, p<0.05). Conversely, when the tones were presented at supra-threshold intensity, subjects perceived more often the first tone as being louder in the passive compared with active condition (passive condition: 46.4±2.3%, active condition: 40.3±2.5%, t_(27)_ = 1.79, p<0.05; Fig 1B).

Additionally, the repeated measures ANOVA revealed a main effect of sound intensity (F_(1,27)_ = 51.161, p<0.001, *η* ^2^_*p*_ = 0.65), reflecting the fact that at near-threshold intensity subjects reported more often the first tone to be louder and at supra-threshold intensity subjects reported more often the second tone to be louder. Further examination revealed that when the tones were presented in near-threshold intensity, the proportion of times that the subjects rated the 1^st^ tone to be louder was significantly above chance, whereas when the tones were presented in supra-threshold intensity, the proportion of times the subjects rated the 1^st^ tone to be louder was significantly below chance (mean percentage of trials ± s.e.m. collapsed across active/passive conditions—near-threshold: 58.4±1.8%, t_(27)_ = 4.9, p<0.0001; supra-threshold: 43.4±1.8%, t_(27)_ = 3.63, p<0.0001).

## Discussion

We demonstrate that when the expected intensity of self-generated tones is very low, perceived loudness is enhanced compared with that of identical tones heard in a passive manner. Conversely, when the expected intensity is high, perceived loudness of self-generated tones is attenuated.

At supra-threshold intensity, our results are in agreement with previous findings showing that the intensity of self-generated tactile [23] and auditory [4,21] stimuli is attenuated. At near-threshold intensity, our results are compatible with studies reporting that self-generated visual [24] and auditory [14,25] stimuli are actually better detected compared with stimuli generated by an external source.

Previous studies examined how perception of self-generated sounds is modulated relative to sounds generated by an external source [4,21,26]. Using pairs of sounds presented at supra-threshold intensities, they report a shift in point-of-subjective-equality (PSE) corresponding to attenuated perception of self-generated sounds. Here, we applied a different experimental design in which subjects were instructed to discriminate the loudness of two tones and used the percentage of “1^st^ tone louder” responses as a dependent measure. Our results at the supra-threshold intensity are compatible with those reported using the PSE measure in previous studies. This suggests that our measure of “1^st^ tone louder” for assessing perceptual differences between self- and externally-generated sounds is valid. Importantly, we examined for the first time also the perceptual modulations following manipulation of sound intensity within the same subjects and find the opposite type of perceptual modulation at near-threshold sound intensities—namely enhancement of self-generated action consequences. The fact that modulation type depends on sound intensity could also explain differences between EEG studies reporting sensory attenuation as reflected in lower N100 amplitude [3], and some fMRI studies reporting sensory enhancement in auditory cortex [27]. Previous EEG studies used stimuli well above hearing threshold in the typically quiet setting (compatible with the current supra-threshold condition). Conversely, the fMRI setup is typically noisy, making auditory stimuli less audible (compatible with the current near-threshold condition).

In the active condition, subjects fully controlled the appearance of the first (self-generated) tone. Therefore, it could be argued that the saliency of self-generated tones (due to their expected appearance) biased subjects’ perceptual judgments. We note that the appearance of all externally-generated tones was temporally cued by a visual stimulus and therefore fully predictable. Nonetheless, differences in predictability between the active and passive conditions cannot be completely ruled out. Importantly, here we found differential modulatory effects across tone intensities—namely enhancement at near-threshold and attenuation at supra-threshold. This argues against differences in expected timing across active/passive trials as an alternative explanation to the differences we find between supra- and near-threshold sound intensity levels.

To conclude, our results suggest that efference signals can modify perception in an adaptive manner that either attenuates or enhances responses to self-generated stimuli according to different sensory contexts. Such a differential functional role is ecologically relevant, as the expected auditory consequence of hammering a nail or clicking the TV remote are not the same. The current results support the notion that during performance of sound-producing actions, an efference copy carries information regarding expected sound intensity and modifies behavioral sensitivity accordingly.
